# Polyphenols Recovery from *Thymus serpyllum* Industrial Waste Using Microwave-Assisted Extraction–Comparative RSM and ANN Approach for Process Optimization

**DOI:** 10.3390/foods11091184

**Published:** 2022-04-19

**Authors:** Živan Mrkonjić, Dušan Rakić, Aleksandar Takači, Muammer Kaplan, Nemanja Teslić, Zoran Zeković, Ivana Lazarević, Branimir Pavlić

**Affiliations:** 1 Faculty of Technology, University of Novi Sad, Bulevar Cara Lazara 1, 21000 Novi Sad, Serbia; zivan_mrkonjic@hotmail.com (Ž.M.); dusan.rakic@uns.ac.rs (D.R.); stakaci@tf.uns.ac.rs (A.T.); zzekovic@tf.uns.ac.rs (Z.Z.); ivana.lazarevic702@gmail.com (I.L.); 2 TUBITAK Marmara Research Centre, Institute of Chemical Technology, P.O. Box 21, Gebze 41470, Kocaeli, Turkey; muammer.kaplan@tubitak.gov.tr; 3 Institute of Food Technology, University of Novi Sad, Bulevar Cara Lazara 1, 21000 Novi Sad, Serbia; nemanja.teslic@fins.uns.ac.rs

**Keywords:** wild thyme, MAE, antioxidant activity, multi-response optimization, artificial neural network, by-product, Lamiaceae

## Abstract

The aim of this study was to valorize *Thymus serpyllum* L. herbal dust, the particular fraction distinguished as industrial waste from filter-tea production. This work demonstrated comparable analysis considering model fitting, influence analysis and optimization of microwave-assisted extraction (MAE) of bioactive compounds from the aforementioned herbal dust using face-centered central composite experimental design within the response surface methodology (RSM), as well as artificial neural networks (ANN). In order to increase yield and amount of compounds of interest and minimize solvent, time and energy consumption, the ethanol concentration (45, 60 and 75%), extraction time (5, 12.5 and 20 min), liquid–solid ratio (10, 20 and 30 mL/g) and irradiation power (400, 600 and 800 W) were used as independent variables. Total extraction yield (Y), total phenols yield (TP), as well as antioxidant activity parameters obtained by DPPH and ABTS assays, were selected as responses. It could be concluded that the MAE technique is an efficient approach for the extraction of biologically active compounds from *T. serpyllum* herbal dust, which represents a high-value source of natural antioxidants with great potential for further use in various forms within different branches of industry.

## 1. Introduction

*Thymus serpyllum* L., known as a highly potent source of bioactive compounds, is widely spread across northern and central Europe. As a perennial shrub, whose herb has been collected and used in many cultures as a healing agent, nowadays mostly as a tea product, it has shown great potential for further research. The first written traces showed that even in ancient Greece, wild thyme was used to treat respiratory and digestive tract [1]. Since then, many more characteristics such as antibacterial, antiviral, astringent, anti-inflammatory, sedative, anticonvulsant, expectorant, antispasmodic, choleretic, analgesic, diuretic, diaphoretic, anticholesterolemic, immunostimulant and anthelmintic have been identified [1,2,3].

It was noted that during the process of production and packing different types of various teas a fraction remained unused. The reason lies in the particle size diameter that is smaller than the pores of filter-tea bags. Although presented as industrial waste, the filter-tea fraction presents a valuable source of polyphenols [4,5,6,7]. Considering the above, wild thyme herbal dust has been chosen as a starting point for the investigation of microwave-assisted extraction (MAE). MAE belongs to green extraction techniques characterized by short extraction time and low energy and solvent consumption [8]. External energy in form of microwaves generates heat energy which enhances the cellular disruption of plant material and accelerates the mass transfer from the matrix into the liquid phase [9]. The heat energy occurs due to dipole rotation and ionic conduction, which is caused by the friction between molecules that rotate and by resistance and friction that appear during ions migration throughout the solution [10].

Parameters that can significantly affect MAE efficiency are the type of solvent, extraction time, liquid–solid ratio, irradiation power, number of cycles, temperature and type of raw material. Polar solvents, i.e., solvents with a high dielectric constant, have an advantage over non-polar ones in terms of better microwave energy adsorption [11]. It must be taken into account that highly polar solvents such as water, due to low microwave dissipation factor, might prevent heating inside the plant matrix and reduce the release efficiency of compounds of interest [12]. For that reason, the mixture of ethanol and water, generally recognized as safe, is considered the first choice for the isolation of compounds of interest using microwave energy [8]. The liquid–solid ratio also plays a significant role in the optimization process of MAE and it should be adjusted in order to entirely soak the plant matrix without leading to a reduction of polyphenols recovery [13]. Temperature has a close connection with irradiation power and extraction time, so an adequate setting of temperature will minimize the risk of thermal degradation and provide extraction of compounds of interest [14]. So far, many studies have successfully conducted microwave-assisted extraction (MAE) in order to maximize the total phenols yield (TP) and antioxidant activity of extracts obtained from various plants from the Lamiaceae family [15,16,17,18].

The main objective of our study was to valorize *T. serpyllum* herbal dust by MAE and to evaluate extraction parameters (ethanol concentration, extraction time, liquid–solid ratio and irradiation power) affecting total extraction yield (Y), total phenols yield (TP) and antioxidant properties of obtained extracts. To our best knowledge, there are no papers investigating the application of response surface methodology (RSM) and artificial neural networks (ANN) on the MAE of *T. serpyllum*. Therefore, RSM and ANN approaches were applied to optimize antioxidants recovery from *T. serpyllum* herbal dust and to conduct comparative analysis in terms of influence analysis, model fitting and optimization accuracy. Finally, validation was performed and qualitative polyphenols profiles of extracts obtained at the central point and under optimal conditions by RSM were determined by HPLC-MS/MS analysis.

## 2. Materials and Methods

### 2.1. Sample

The sample was kindly donated by Macval D.O.O. Novi Sad (Serbia) and it is considered a representative sample of the population that unites plants grown and collected in the territory of the Republic of Serbia. Due to its mean particle size of less than 0.315 mm and the impracticality of having this fraction packed in the filter-tea bag, it is characterized as a by-product. The *T. serpyllum* herbal dust was stored in a paper bag at room temperature prior to extractions.

### 2.2. Chemicals

Folin–Ciocalteu reagent, (±)-6-hydroxy-2,5,7,8-tetramethylchromane-2-carboxylic acid (Trolox) (≥98%), 2,2-diphenyl-1-picrylhydrazyl (DPPH) (≥90%), 2,4,6-tris(2-pyridyl)-s-triazine (≥99.0%) and gallic acid (≥98.0%) were supplied from Sigma-Aldrich (Steinheim, Germany). 2,2′-azino-bis(3-ethylbenzothiazoline-6-sulfonic acid) diammonium salt (ABTS) (98%) was purchased from J&K, Scientific Ltd., Beijing, China. Additionally, ferric chloride hexahydrate (>99%) and sodium carbonate anhydrous (>99%) were supplied from Centrohem, Stara Pazova, Serbia, while potassium peroxydisulfate (>99.6%) and acetic acid (99.8%) were purchased from Lach-Ner, Neratovice, the Czech Republic. Sodium acetate anhydrous (>99%) was purchased from Kemika, Zagreb, Croatia. The ultra-pure water was obtained by a Milli-Q Plus system (EMD Millipore, Billerica, MA, USA).

### 2.3. Microwave-Assisted Extraction (MAE)

MAE was conducted in a homemade open microwave system consisting of a microwave oven (MM817ASM, Bosch, Gerlingen, Germany), a round glass flask (500 mL) and a condenser. The MAE experiments were performed in a single mode at a fixed frequency of 2.45 GHz, where the temperature varied depending on the boiling point of the mixture of raw material and applied solvent. The glass flask was fixed at the same position as the microwave system and no additional agitation was applied. Obtained crude extracts were cooled down, filtered through filter paper under vacuum (V-700, Büchi, Flawil, Switzerland), collected into plastic vials and stored at 4 °C prior to analysis.

### 2.4. Determination of Y and TP

Using the rotary vacuum evaporator (RV 05, IKA, Staufen im Breisgau, Germany) 10 mL of crude extract was evaporated under 45 °C and further dried in the oven at 105 °C until constant mass. Results of Y were presented as a mass of total extractable solids per 100 g of dry plant material (%; *w/w*). Determination of TP of obtained extracts was performed by Folin–Ciocalteu assay [19]. Absorbances were recorded at 750 nm using a spectrophotometer (Jenway, model 6300, Stone, UK). All experiments were performed in triplicate and mean values of TP of obtained extracts were presented as grams of gallic acid equivalent (GAE) per 100 g of sample dry weight (g GAE/100 g).

### 2.5. Antioxidant Activity of Extracts

Antioxidant activity of extracts was determined by spectrophotometric methods towards DPPH [20] and ABTS radicals [21], as well as by the ferric ion reducing antioxidant power (FRAP) assay [22]. DPPH assay was performed by mixing 100 μL of extract with 2900 μL of DPPH solution, which was previously prepared at a concentration of 26 mg/L of methanol. After 1 h in the dark at room temperature, the absorbances were recorded at a wavelength of 517 nm. ABTS assay was performed by mixing 100 µL of extract with 2900 µL of ABTS reagent. After 5 h in the dark at room temperature, the absorbances were recorded at a wavelength of 734 nm. The FRAP assay was performed by mixing 100 µL of extract with 2900 µL of FRAP reagent. After incubation in the dark at 37 °C for 10 min, the absorbances were recorded at a wavelength of 593 nm. All experiments were performed in triplicate and mean values were presented in the case of DPPH and ABTS assays as mM of Trolox equivalent (TE) per gram of sample dry weight (mM TE/g) and in the case of FRAP assay mM of Fe^2+^ per gram of sample dry weight (mM Fe^2+^/g).

### 2.6. Q Exactive Hybrid Quadrupole-Orbitrap LC-MS/MS Analysis

With the aim of testing free, ester-linked and glycoside-linked phenolic compounds in samples, the Q Exactive LC-MS/MS–Orbitrap (Thermo Scientific, Hemel Hempstead, UK) was employed. The same method was applied and described in detail in the study by Pavlić et al. [17]. Chromatographic separation of compounds was achieved on a Poroshell 120 EC-C18 column (3.0 × 100 mm, 2.7 µm, Agilent) setting gradient flow at 0.6 mL/min (mobile phase A: 0.1% formic acid–water, mobile phase B: methanol; 0–5 min, mobile phase B concentration changed as 0–9% B; 5–9 min, 9–2% B; 16–35 min, 2–18% B; 35–50 min, 18–20% B; 50–65 min, 20–30% B and 65–80 min, 30% B). The injection volume was 10 µL. A Q Exactive hybrid quadrupole-Orbitrap mass spectrometer equipped with an ESI source working in both negative and positive ionization mode was used for accurate mass measurements. The following parameters were set: ion spray voltage, 2.8 kV; capillary temperature, 300 °C; capillary voltage, 35 V and tube lens voltage, 95 V; sheath gas, 19 (arbitrary units); auxiliary gas, 7 (arbitrary units). Mass spectra were recorded covering the m/z range of 55–1000 Da. Default values were used for most other acquisition parameters (Automatic gain control (AGC) target 3 × 10^6^ ions). The data processing was achieved using XCalibur 2.2 software (Thermo Fisher Scientific, Waltham, MA, USA). An external calibration for mass accuracy was performed before the analysis.

### 2.7. Design of Experiments and Statistical Methods

With the aim of investigating the impact of the MAE parameters on target responses and optimizing the extraction process, response surface methodology (RSM) and artificial neural networks (ANNs) were used. The impacts of ethanol concentration (45, 60 and 75%), extraction time (5, 12.5 and 20 min), liquid–solid ratio (10, 20 and 30 mL/g) and irradiation power (400, 600 and 800 W) were used as input parameters in both methods. A study of Mrkonjić et al. [23], that investigated the impact of different concentrations of ethanol on Y, TP and DPPH using conventional solid–liquid extraction of *T. serpyllum* herbal dust, made it easier to define an appropriate domain of concentration of ethanol. Extraction time and liquid–solid ratio were set by the pattern of similar studies, while irradiation power was based on the capabilities of the microwave oven [17,18,24].

#### 2.7.1. Response Surface Methodology (RSM)

Under the RSM, the face-centered central composite experimental design (CCD), which included four input parameters arranged at three levels, was used. Taking into account five replicates at the central point, it consisted of a total of 29 runs. Multiple regression was applied and the results were fitted to a second-order polynomial model (Equation (1)).

(1) $$Y={\;\beta }_{0}+\overset{4}{\underset{i=1}{\sum }}\beta_{i}X_{i}+\overset{4}{\underset{i=1}{\sum }}\beta_{\mathrm{ii}}X_{i}^{2}+\overset{3}{\underset{i=1}{\sum }}\overset{4}{\underset{j=i+1}{\sum }}\beta_{\mathrm{ij}}X_{i}X_{j}\;$$

*Y* represents the response variable, X_i_ and X_j_ are the independent variables affecting the response, and β_0_, β_i_, β_ii_, and β_ij_ are the regression coefficients for intercept, linear, quadratic and cross-product terms. The goodness of fit was determined by analysis of variance (ANOVA), while model adequacy was evaluated by the coefficient of determination (*R*^2^), coefficient of variance (CV) and *p*-values for the model and lack of fit. Optimal extraction conditions were determined considering desired Y and TP, as well as antioxidant activity parameters obtained by DPPH and ABTS assays. The selection of optimal conditions was based on the desirability function (*D*) [25]. In order to verify obtained empirical models, validation was performed by using the extracts prepared at optimal conditions determined by the selected statistical methodologies. For the listed analysis, Design-Expert v.11 software (Stat-Ease, Minneapolis, MN, USA) was used with a significance level of 0.05.

#### 2.7.2. Artificial Neural Network (ANN)

Constructed ANN models consisted of four input parameters and one output, which means that each model individually for each investigated response was developed. The input layer consisted of 4 nodes representing the input parameters, 1 hidden layer with 5–10 nodes and the output layer consisted of 1 node representing the response. The number of hidden nodes differs in each network as the node number is a subject of optimization. For training, 70% of data was used, 15% for testing and 15% for validation. The training process consisted of three steps, where in the first step feedforward of the input training pattern was performed. In the second step, calculation and backpropagation of the associated error were conducted and finally, the gradient-dependent adjustment of the calculated weights was conducted. ANN calculations were performed using Statistica 10 software package.

Error estimation was achieved by the Symbiotic organisms search (SOS) method and the training algorithm was achieved by the Broyden–Fletcher–Goldfarb–Shanno (BFGS) algorithm. For the error measurement, the *R*^2^ quotient was used. Out of 20 networks for each output, the best were chosen based on the *R* values and a global sensitivity analysis was performed for each network. The optimization was based according to validation error minimization. In order to find the optimal values of input parameters, a new neural network was constructed with 4 input and 4 output nodes and one hidden layer. The inputs and outputs were normalized. The sum of normalized outputs was the value that was maximized in the optimization process.

## 3. Results and Discussion

### 3.1. Total Extraction Yield (Y) and Total Phenols Yield (TP)

Experimentally observed values of Y and TP according to face-centered CCD, including four input parameters (ethanol concentration, extraction time, liquid–solid ratio and irradiation power) on three levels, are presented in Table 1. In order to normalize independent variables, their actual values are presented in the form of coded values that were set by the range from −1 to 1.

According to Table 1, Y was in the range 10.69–17.60%, where the lowest value of Y was observed using 75% of ethanol, liquid–solid ratio of 10 mL/g at 400 W for 5 min (run 2). On the other hand, using 60% of ethanol, liquid–solid ratio of 20 mL/g at 600 W for 12.5 min led to highest value of Y (run 25). Recovery of polyphenols was the lowest (2.8903 g GAE/100 g DW) by using 45% of ethanol, liquid–solid ratio of 30 mL/g at 400 W for 20 min (run 7). Even though the recovery of polyphenols was the highest using 45% of ethanol, but with a different liquid–solid ratio (10 mL/g) at 400 W for 20 min (4.2637 g GAE/100 g DW; run 3), the lower liquid–solid ratio is desirable in the sense of preventing excessive dilution of liquid extracts and decreasing cost of their further processing [17]. In addition, an observation that the highest values of TP were obtained using 50% of ethanol concentration was noticed by Jovanović et al. [26], who investigated maceration, heat-assisted and ultrasound-assisted extractions (UAE) of *T. serpyllum*. In comparison with ultrasound-assisted and pressurized-liquid extraction (PLE), it could be seen that MAE provided particularly lower Y values, values that are close to those obtained by conventional extraction [23,27]. On the other hand, observed high values of TP and lower values of Y indicate that MAE could be considered a good approach for the selective isolation of polyphenols from *T. serpyllum*, facilitating the downstream process.

The significance of the influence of input parameters and their interactions on target responses are presented in Table 2.

According to *p*-values obtained by ANOVA analysis, linear terms of ethanol concentration and liquid–solid ratio exhibited a highly significant effect (*p* < 0.01) on Y and TP, while the linear term of extraction time had a moderately significant effect (0.01 < *p* < 0.05) only on Y. Ethanol concentration had a negative influence on Y (Figure S1a), but in the case of TP, it was in the range of experimental data and its maximum value was obtained at an ethanol concentration of 55.22%, after which it had more intensive negative influence (Figure S2a). The liquid–solid ratio had a negative influence on Y (Figure S1b), but positive on TP (Figure S2b). The same trend with ethanol concentration was reported by Zeković et al. [17], whose investigation was based on UAE and MAE optimization of *Salvia officinalis* L. herbal dust. Linear term of irradiation power exhibited almost negligible influence (Figure S2d), but extraction time provided a slightly positive one on TP (Figure S2c). By increasing the irradiation power, the Y value at one point reaches a maximum after which it begins slightly to decline (Figure S1d). On the other hand, increasing the extraction time does not necessarily mean that the extraction of polyphenols will be efficient, due to the possibility of potential extraction of non-target compounds. In addition, prolonged time in combination with microwave energy could enhance the denaturation of the plant cell walls and accelerate the diffusion of the aforementioned compounds to the liquid phase [28,29]. The influence of interactions was noticed as moderately significant only between extraction time and liquid–solid ratio on Y, as well as between ethanol concentration and liquid–solid ratio on TP. The influence of ethanol concentration quadratic term was pronounced in the case of TP as highly significant and moderately significant in the case of Y. Although the influence of ethanol concentration was not in the range of experimental data, the explanation for this lies in the occurrence of potential non-selective extraction, as well as in the technical difficulties during the filtration process of the obtained crude extract [23].

### 3.2. Antioxidant Activity of T. serpyllum Extracts

The antioxidant activity of *T. serpyllum* extracts was assessed through DPPH, FRAP and ABTS tests. Polyphenols free radicals, which arise by hydrogen or electron transfer reactions, were measured using DPPH and ABTS antioxidant capacity. On the other hand, the reducing power of extracts was measured using the FRAP test, due to the capability of polyphenols to prevent oxidation by chelation and reduction of prooxidant metals and inhibition of oxidizing enzymes [30,31]. The experimental values, obtained by the already mentioned tests, are presented in Table 1. The lowest scavenging capacity of DPPH (0.1567 mM TE/g) was observed in run 7 (45% ethanol, 20 min, 30 mL/g and 400 W), while the highest one (0.2382 mM TE/g) was observed in run 18 using the following values of input parameters, 75% ethanol, 12.5 min, 20 mL/g and 600 W. In the case of FRAP, the lowest value was observed in run 4 (0.3590 mM Fe^2+^/g) using 75% of ethanol, 20 min extraction time, 10 mL/g liquid–solid ratio and irradiation power of 400 W. Using 45% of ethanol, 12.5 min extraction time, 20 mL/g liquid–solid ratio and irradiation power of 600 W the highest value of FRAP (0.7182 mM Fe^2+^/g) was accomplished (run 17). Extract obtained in run 24 (60% ethanol, 12.5 min, 20 mL/g and 800 W) exhibited the lowest scavenging capacity of free ABTS radicals (0.3284 mM TE/g), while the highest one (0.5120 mM TE/g) was exhibited in run 19 using 60% of ethanol, liquid–solid ratio of 20 mL/g at 600 W for 5 min. Obtained values were in accordance with the results provided by Mrkonjić et al. [23], who investigated the antioxidant activity of UAE wild thyme herbal dust extracts using the same tests. Ðukić et al. investigated the antioxidant activity of *T. serpyllum* extracts obtained by MAE (96% ethanol, 30 min, 20 mL/g, 600 W). However, obtained results were expressed as IC_50_ (29.60 mg/mL), which could not be compared with results obtained by the present study [32].

Linear terms of ethanol concentration and extraction time exhibited a highly significant influence on DPPH, while the linear term of the liquid–solid ratio showed a significant impact on ABTS (Table 2). Only a combination of ethanol concentration and liquid–solid ratio showed moderate significance on DPPH, while all other interaction terms did not show a significant impact on other responses. The quadratic term of ethanol concentration exhibited a moderately significant impact on ABTS, the liquid–solid ratio the same on DPPH, while irradiation power showed a significant impact on FRAP. Taking into account the aforementioned quadratic terms and responses, ABTS, DPPH and FRAP values were in the range of experimental data. Positive or negative influence of input parameters on DPPH, FRAP and ABTS was presented in Figures S3, S4 and S5, respectively. Due to the significant impact of the quadratic term (Table 2), it could be observed that ethanol concentration had a positive influence on ABTS until reaching the ethanol concentration of 57.41% (Figure S5a), after which the impact started to decline. Considering that antioxidant activity and TP are closely related, it could be noticed that the same trend with ethanol concentration was observed by the finding of Simić et al. [24], whose goal was to maximize TP of *Aronia melanocarpa* MAE extracts. This could be explained by increasing the dipole moment of the solvent throughout higher absorption of microwaves. Moreover, such a kind of solvent shows a higher affinity towards moderately polar polyphenols in terms of enhanced diffusion of the solvent into the plant cells, which leads to better heating and facilitates the mass transfer from solid to liquid phase [29,33].

### 3.3. RSM versus ANN Model Fitting

Adequacy and statistical significance of the applied quadratic model obtained by RSM were checked by analysis of variance (ANOVA) (Table 3). Based on values of *R^2^* obtained for Y, TP, DPPH, FRAP and ABTS (0.9242, 0.8487, 0.9216, 0.4733 and 0.6661, respectively), it could be concluded that there is a good fit between experimentally observed and predicted values, except in the case of FRAP and ABTS.

The values of coefficient of variance (CV), that describe the dispersion degree of the data, for Y, TP, DPPH, FRAP and ABTS, were 4.11, 4.55, 4.14, 12.57 and 8.44%, respectively. Those values lead to the conclusion that there is a good fitness of the applied model, but again except in the case of FRAP and ABTS. The lack of fit for all responses excluding FRAP was not statistically significant (*p* > 0.05). Considering the above, only FRAP was excluded in the process of both RSM and ANN optimization.

**Figure 1:**
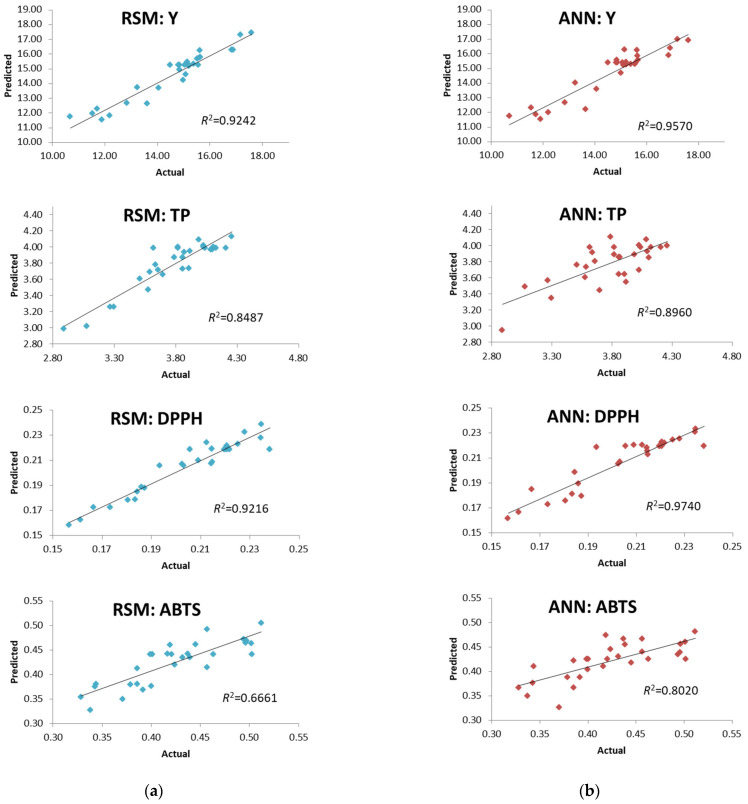
Comparison of experimentally observed and predicted values determined by (**a**) RSM and (**b**) ANN.

Recently, several studies have focused on the comparative analysis of RSM and ANN modeling applied to the optimization of polyphenols recovery from various plant resources such as blackcurrants [34], Meghalayan cherry fruit [35] and kidney beans [36]. However, Teslić et al. [30] recently proposed a comprehensive comparison of these statistical tools applied to the optimization of polyphenols isolation from defatted wheat germ. Therefore, the comparison between RSM and ANN approaches in this work was performed on three levels, where in the first one the goodness of fit was evaluated. Considering obtained regression plots and their coefficients, it has been noticed that corresponding coefficients for ANN were slightly higher than those obtained for RSM (Figure 1). This is in accordance with results published by Teslić et al. [30].

Fitting of experimental results by RSM was done using second-order polynomial model. On the other hand, fitting of experimental results by ANN was done using nonlinear models. Due to potential “over-fitting”, ANN simulation was realized with 5 to 10 neurons in the hidden layer. Summary of active networks and their training and test performances were presented in Table S1. Considering particularly high values of *R*^2^ for Y, TP, DPPH and ABTS (0.9570, 0.8960, 0.9740 and 0.8020, respectively), it could be concluded that ANN provided adequate fit of experimental data for aforementioned responses (Figure 1b).

**Figure 2:**
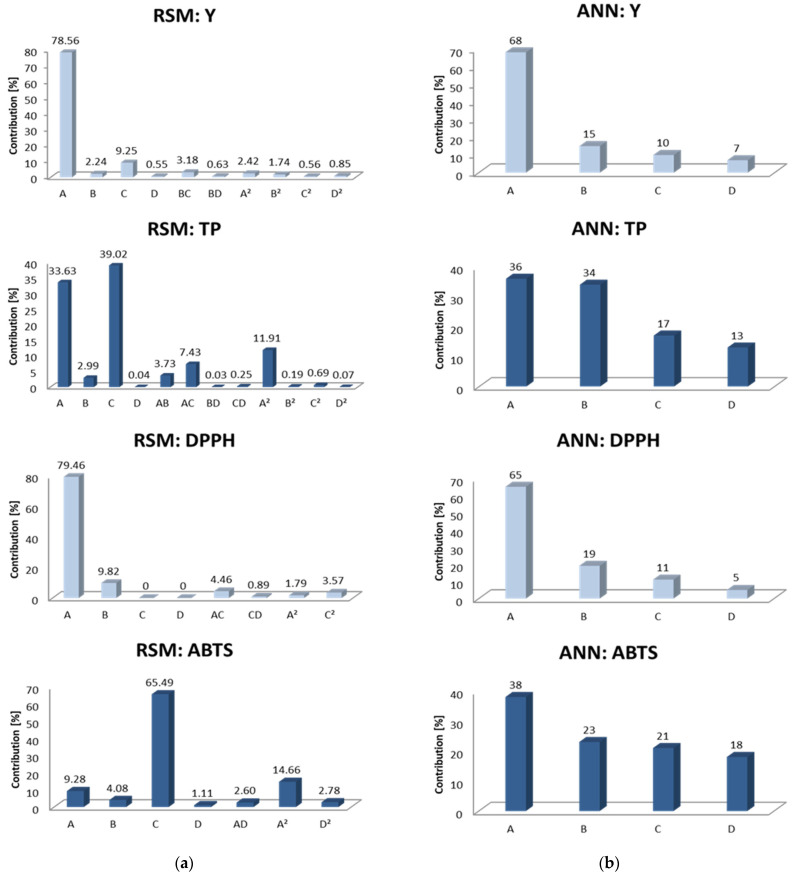
Contributions of ethanol concentration (A), extraction time (B), liquid–solid ratio (C) and irradiation power (D) observed by (**a**) RSM and (**b**) ANN.

The second level of comparison between RSM and ANN was evaluated through contributions of input parameters (Figure 2).

In consideration of contributions of linear, interaction and quadratic terms obtained by RSM and contributions obtained by sensitivity analysis throughout the ANN approach, it could be concluded that ethanol concentration was the most significant parameter that influenced Y, TP and DPPH, except in the case of ABTS, where the liquid–solid ratio was treated as the most significant parameter (Figure 2a). On the other hand, ANN showed that ethanol concentration was the most significant parameter in the case of all responses (Figure 2b). This is in agreement with the finding by Mrkonjić et al. [23], who investigated the significance of UAE parameters on the same target responses. However, another study by Mrkonjić et al. [27], which covered the PLE of *T. serpyllum* herbal dust, singled out ethanol concentration as the third most significant parameter, after temperature and extraction time. According to Veggi et al. [33], the most important factor that affects MAE efficiency is solvent choice. Furthermore, solvents used in conventional extractions could differ in terms of the capacity of the solvent to absorb the microwave energy and heat up. Besides solvent, the liquid–solid ratio is considered the second most important parameter affecting MAE efficiency. The reason for that lies in more energy consumption when a high liquid–solid ratio is used. In addition, the inadequate setting of this parameter could require additional time downstream of the process and cause nonuniform distribution and exposure to microwaves [13,33].

For all responses, ANN has identified extraction time as the second, liquid–solid ratio as the third, and irradiation power as the fourth most significant parameter. RSM provided the same influence as ANN on DPPH, while the liquid–solid ratio was the second, extraction time the third and irradiation power the fourth most significant parameter that affected Y and TP. In the case of RSM and contributions obtained for ABTS, the significance of parameters was as follows: liquid–solid ratio > ethanol concentration > extraction time > irradiation power. However, the significance of irradiation power was almost negligible, which was not the case with ANN, where the contribution was in the range of 5 to 18% (Figure 2).

The third level of comparison between RSM and ANN was evaluated considering experimentally observed and predicted values obtained after the optimization process by RSM and ANN, which was explained in Section 3.4. However, given the accuracy of the methodology, the ANN approach has a slight advantage over RSM. The same trend was noticed in the finding of Simić et al. [24], who used RSM and ANN for modeling and optimizing the MAE of TP from *Aronia melanocarpa*.

According to the significance of linear, cross-product and quadratic terms on all responses excluding FRAP, the predictive model equations are presented in Table 4.

### 3.4. Process Optimization and Experimental Verification

The planned experiment allowed us to realize the process optimization and to find the optimal working conditions using both RSM and ANN. All responses except FRAP were taken into account in the optimization process, where the goal of optimization was to maximize all of them. Optimized conditions obtained by RSM were the ethanol concentration of 52.19%, extraction time of 20 min, liquid–solid ratio of 23.64 mL/g and irradiation power of 400 W, with a desirability function of 0.88. On the other hand, optimized conditions obtained by ANN were the ethanol concentration of 45.00%, extraction time of 5 min, a liquid–solid ratio of 30.00 mL/g and the same value of irradiation power, as in the case of RSM. The optimization process of ANN was performed in C++ programming language by the brute force method, which ran all input values with the increment of 0.01. Predicted values for ANN were calculated using developed mathematical models by Statistica software, while in the case of RSM predicted values were obtained based on quadratic model equations given in Table 4. The same methodology was applied in our previous research focused on optimization of the extraction process by RSM and ANN approaches [30]. According to obtained optimal conditions, the experimental verification was conducted (Table 5).

It could be concluded that both RSM and ANN approaches showed satisfactory correlation between predicted and experimental values. Despite the different conditions under which both verifications were performed, the experimentally obtained values were high and relatively close to each other. Furthermore, both approaches could be recognized as an infallible choice for MAE optimization of *T. serpyllum* herbal dust. According to the techno-economical aspect, a slight preference is given to ANN, where a high-quality extract would be produced in the shortest possible time.

### 3.5. Polyphenols Profile

The qualification of polyphenols in extracts obtained on the central point (Sample MAE-CP) and under the optimal conditions (Sample MAE-OPT) was carried out using Q Exactive hybrid quadrupole-orbitrap LC-MS/MS analysis (Table 6). A total of 35 different compounds were detected.

Detected phenolic acids in the extract obtained on the central point were gallic, vanillic, protocatechuic, 3-*p*-coumaroylquinic, 4-*p*-coumaroylquinic, *p*-coumaric acid, coumaric acid hexoside isomer-1, coumaric acid hexoside isomer-2, coumaric acid hexoside isomer-3, *cis*- and *trans*-coutaric, caffeic and ellagic acid. In extract obtained under optimal conditions all aforementioned phenolic acids were identified except for *cis*- and *trans*-coutaric acid. In comparison with the present study, Boros et al. [37] have additionally identified chlorogenic, ferulic and rosmarinic acids in methanol extracts of *T. serpyllum*, whereas the raw material originated from Hungarian and Romanian habitats.

Compounds that were identified in both extracts were dihydroxycoumarin, quercetin in form of hexoside isomer-1, hexoside isomer-2, 3-*O*-galactoside, 3-*O*-glucoside, 3-*O*-rutinoside and glucuronide, kaempferol, kaempferol-3-rutinoside, 3-galactoside and 3-glucoside, naringenin-7-*O*-glucoside, isorhamnetin-3-*O*-galactoside, eriodictyol and luteolin. Furthermore, monogalloyl-glucose, a compound that belongs to the tannin group, was identified in both extracts as well. However, (+)-catechin and (-)-epicatechin together with naringenin were identified only in extract MAE-OPT. On the other hand, quercetin in a free form and its derivatives, quercetin pentoside isomer-1 and quercetin pentoside isomer-2, were identified only in extract MAE-CP.

In the study of Ivasenko et al. [3], although based on UAE of bioactive compounds from the population of *T. serpyllum* from Central Kazahstan, phenolic acids and flavonoids prevailed in the extracts obtained with 70% of ethanol, a liquid–solid ratio of 1:20 at room temperature for 30 min. Among the contents of extracts, the following bioactive compounds stood out: luteolin-7-*O*-glucoside, rosmarinic acid, naringenin, epicatechin, catechin, myricetin, quercetin, apigenin, kaempferol, caffeic, gallic, chlorogenic, ferulic acid, rutin and luteolin. According to Fecka and Turek [38], in methanolic extract of *T. serpyllum* caffeic acid and its derivatives, eriocitrin, luteolin, luteolin-7-*O*-rutinoside, luteolin-7-*O*-glucuronide, eriodictyol, lithospermic and rosmarinic acid were detected. Considering the influence of the locality of origin, environmental and other conditions, as well as extraction process and all factors included, a certain similarity among differently obtained extracts could be recognized. It may be concluded that all the aforementioned could greatly affect the chemical composition and yield of the *T. serpyllum*. For that reason, the qualitative profile of the extract is of great importance, with the purpose of its further use in the pharmaceutical, cosmetic and food industries.

## 4. Conclusions

In comparison with ANN, RSM could provide further insights into the influence analysis of cross-product and quadratic terms. Moreover, RSM could also provide a more detailed graphical visualization of the influence of input parameters on responses. Both techniques suggested that ethanol concentration was the most significant factor affecting all responses, except in the case of RSM on ABTS, where the liquid–solid ratio was the most significant parameter. Optimal conditions obtained by RSM were the ethanol concentration of 52.19%, extraction time of 20 min, a liquid–solid ratio of 23.64 mL/g and irradiation power of 400 W, while optimal conditions obtained by ANN were an ethanol concentration of 45.00%, extraction time of 5 min, liquid–solid ratio of 30.00 mL/g and irradiation power of 400 W. Verification demonstrated that both RSM and ANN approaches showed good correlation between predicted and experimental values. Considering the fitting quality, influence analysis and optimization, both mathematical procedures RSM and ANN could be successfully used in order to describe experimental data. According to the techno-economical aspect, a slight preference is given to ANN, where, by reducing extraction time, the quality of polyphenol-enriched wild thyme MAE extract may remain unchanged. It could be also concluded that MAE represents a cost-effective approach for the isolation of natural bioactive compounds from *T. serpyllum* herbal dust. Furthermore, it could be used in a form of liquid or powder extract in the agri-food sector, pharmaceutical and cosmetic industry as a substitution for synthetic products. Liquid extracts obtained by MAE will be processed into powder form by spray drying technology. Further research on wild thyme herbal dust extracts will include supercritical fluid extraction of lipophilic bioactive compounds and evaluation of their chemical profile and bioactivity. The perspective of the application of wild thyme powder and lipophilic extracts as natural additives in food products such as meat and dairy products will be determined in our following studies.

## Figures and Tables

**Table 1:** Face-centered CCD with coded and actual values of input parameters and experimentally observed values of investigated responses.

Run	Input Parameters								Responses				
	A: Ethanol Concentration (%)		B: Extraction Time (min)		C: Liquid–Solid Ratio (mL/g)		D: Irradiation Power (W)		Y (%)	TP (g GAE/100 g DW)	DPPH (mM TE/g)	FRAP (mM Fe^2+^/g)	ABTS (mM TE/g)
1	45	−1	5	−1	10	−1	400	−1	15.21	3.8200	0.2027	0.5945	0.4570
2	75	1	5	−1	10	−1	400	−1	10.69	3.9155	0.1861	0.5763	0.4373
3	45	−1	20	1	10	−1	400	−1	15.00	4.2637	0.2145	0.6395	0.5018
4	75	1	20	1	10	−1	400	−1	13.25	3.2721	0.1836	0.3590	0.3857
5	45	−1	5	−1	30	1	400	−1	14.06	3.2983	0.1734	0.5445	0.3789
6	75	1	5	−1	30	1	400	−1	14.50	4.1299	0.2057	0.6792	0.4208
7	45	−1	20	1	30	1	400	−1	11.54	2.8903	0.1567	0.4678	0.3377
8	75	1	20	1	30	1	400	−1	15.66	4.0896	0.2033	0.6032	0.4970
9	45	−1	5	−1	10	−1	800	1	15.54	4.1036	0.2150	0.6932	0.4163
10	75	1	5	−1	10	−1	800	1	14.86	4.0438	0.2208	0.7071	0.5023
11	45	−1	20	1	10	−1	800	1	11.72	3.5056	0.1666	0.5372	0.4318
12	75	1	20	1	10	−1	800	1	15.10	4.0288	0.2148	0.6319	0.4942
13	45	−1	5	−1	30	1	800	1	12.85	3.0775	0.1613	0.4854	0.3707
14	75	1	5	−1	30	1	800	1	15.62	3.9141	0.2091	0.5634	0.3918
15	45	−1	20	1	30	1	800	1	11.90	3.6965	0.1806	0.5713	0.4237
16	75	1	20	1	30	1	800	1	12.20	3.8593	0.1845	0.5768	0.4393
17	45	−1	12.5	0	20	0	600	0	13.64	3.5796	0.1874	0.7182	0.3438
18	75	1	12.5	0	20	0	600	0	14.82	4.2085	0.2382	0.4889	0.4634
19	60	0	5	−1	20	0	600	0	15.39	4.1064	0.2208	0.7109	0.5120
20	60	0	20	1	20	0	600	0	15.06	3.6245	0.2199	0.6465	0.4009
21	60	0	12.5	0	10	−1	600	0	15.58	3.8154	0.2197	0.4470	0.3991
22	60	0	12.5	0	30	1	600	0	16.84	3.9876	0.2125	0.4525	0.4960
23	60	0	12.5	0	20	0	400	−1	14.86	4.0288	0.2208	0.4416	0.4453
24	60	0	12.5	0	20	0	800	1	16.90	3.6371	0.2218	0.5167	0.3284
25	60	0	12.5	0	20	0	600	0	17.60	3.6558	0.2346	0.5725	0.3855
26	60	0	12.5	0	20	0	600	0	15.21	3.8706	0.2253	0.5196	0.4193
27	60	0	12.5	0	20	0	600	0	15.15	3.5922	0.1936	0.5549	0.3429
28	60	0	12.5	0	20	0	600	0	15.64	3.7930	0.2280	0.4882	0.4570
29	60	0	12.5	0	20	0	600	0	17.19	3.8580	0.2348	0.5786	0.4000

**Table 2:** Significance of linear, cross-product and quadratic terms on Y, TP, DPPH, FRAP and ABTS.

Coefficients	Y	TP	DPPH	FRAP	ABTS
	*p*-Value				
Linear					
X_1_-Ethanol concentration	<0.0001 *	0.0001 *	<0.0001 *	0.3235	0.0616
X_2_-Extraction time	0.0448 **	0.1554	0.0014 *	0.6279	0.2024
X_3_-Liquid–solid ratio	0.0004 *	<0.0001 *	0.6736	0.0339	<0.0001 *
X_4_-Irradiation power	0.2989	0.8638	0.5279	0.0545	0.5151
Cross-product					
X_1_X_2_	-	0.1157	0.6323	-	-
X_1_X_3_	-	0.0321 **	0.0161 **	-	-
X_1_X_4_	-	-	0.5840	-	0.3010
X_2_X_3_	0.0191 **	-	0.6873	-	-
X_2_X_4_	0.2665	0.8809	-	0.2744	-
X_3_X_4_	-	0.6695	0.2751	-	-
Quadratic					
X_1_^2^	0.0377 **	0.0090 *	0.1226	-	0.0216 **
X_2_^2^	0.0729	0.7068	0.6754	-	-
X_3_^2^	0.2946	0.4842	0.0311 **	-	-
X_4_^2^	0.1989	0.8243	-	0.0098 *	0.2836

* *p* < 0.01—significant, ** 0.01 < *p* < 0.05—moderately significant.

**Table 3:** ANOVA and descriptive statistics parameters for applied quadratic models.

Response	Source	Sum of Squares	df	Mean Square	*F*-Value	*p*-Value
Y	Model	79.21	10	7.92	21.94	<0.0001
	Residual	6.5	18	0.361		
	Lack of Fit	5.86	14	0.4187	2.64	0.1804
	Pure Error	0.6355	4	0.1589		
	Cor Total	85.71	28			
	*R*^2^ = 0.9242					
	CV = 4.11%					
TP	Model	2.66	12	0.2218	7.48	0.0002
	Residual	0.4744	16	0.0296		
	Lack of Fit	0.2434	12	0.0203	0.3513	0.9284
	Pure Error	0.231	4	0.0577		
	Cor Total	3.14	28			
	*R*^2^ = 0.8487					
	CV = 4.55%					
DPPH	Model	0.0135	12	0.0011	15.68	<0.0001
	Residual	0.0011	16	0.0001		
	Lack of Fit	0.0006	12	0.0001	0.383	0.9114
	Pure Error	0.0005	4	0.0001		
	Cor Total	0.0146	28			
	*R*^2^ = 0.9216					
	CV = 4.14%					
FRAP	Model	0.1038	6	0.0173	3.29	0.0182
	Residual	0.1155	22	0.0052		
	Lack of Fit	0.1123	18	0.0062	7.8	0.0298
	Pure Error	0.0032	4	0.0008		
	Cor Total	0.2193	28			
	*R*^2^ = 0.4733					
	CV = 12.57%					
ABTS	Model	0.0535	7	0.0076	5.99	0.0006
	Residual	0.0268	21	0.0013		
	Lack of Fit	0.0188	17	0.0011	0.5563	0.8241
	Pure Error	0.008	4	0.002		
	Cor Total	0.0803	28			
	*R*^2^ = 0.6661					
	CV = 8.44%					

df—degrees of freedom.

**Table 4:** Predictive model equations in coded values for total extraction and phenols yield and antioxidant activity obtained by RSM.

Response	Model Equation
Y	$\begin{array}{c} Y=15.2400-1.8100X_{1}+0.3053X_{2}-0.6210X_{3}+0.1515X_{4}-0.3865X_{2\;}X_{3}\\ -0.1722X_{2}X_{4}-0.8383X_{1}^{2}+0.7117X_{2}^{2}-0.4033X_{3}^{2}-0.4983X_{4}^{2} \end{array}$
TP	$\begin{array}{c} \mathrm{TP}=3.9900-0.2028X_{1}+0.0605X_{2}+0.2184X_{3}-0.0071X_{4}+0.0716X_{1\;}X_{2}\\ +0.1011X_{1}X_{3}-0.0066X_{2}X_{4\;}-0.0187X_{3}X_{4}-0.3184X_{1}^{2}\\ +0.0410X_{2}^{2}-0.0767X_{3}^{2}+0.0242X_{4}^{2} \end{array}$
DPPH	$\begin{array}{c} \mathrm{DPPH}=0.2185-0.0222X_{1}+0.0077X_{2}+0.0009X_{3}+0.0013X_{4}+0.0057X_{1}X_{3}\\ -0.0024X_{3}X_{4}-0.0082X_{1}^{2}-0.0119X_{3}^{2} \end{array}$
FRAP	$\begin{array}{c} \mathrm{FRAP}=0.6253-0.0172X_{1}-0.0084X_{2}+0.0386X_{3}-0.0347X_{4}-0.0203X_{2\;}X_{4}\\ -0.0785X_{4}^{2} \end{array}$
ABTS	$\begin{array}{c} \mathrm{ABTS}=0.4400-0.0166X_{1}+0.0111X_{2}+0.0443X_{3}-0.0056X_{4}+0.0095X_{1\;}X_{4}\\ -0.0480X_{1}^{2}+0.0213X_{4}^{2} \end{array}$

X_1_—Ethanol concentration; X_2_—Extraction time; X_3_—Liquid–solid ratio; X_4_—Irradiation power.

**Table 5:** Predicted and experimental values of investigated responses obtained at optimal conditions according to RSM and ANN optimization.

**Input Parameters**	**RSM**		**ANN**	
	**Optimal Parameters**			
Ethanol concentration [%]	52.19		45	
Extraction time [min]	20		5	
Liquid–solid ratio [mL/g]	23.64		30	
Irradiation power [W]	400		400	
**Output Parameters**	**Predicted Values**	**Experimental Values**	**Predicted Values**	**Experimental Values**
Y [%]	16.07	16.89	15.68	17.46
TP [g GAE/100 g]	4.16	4.86 ± 0.1830	4.54	4.36 ± 0.1556
DPPH [mM TE/g]	0.23	0.26 ± 0.0039	0.23	0.20 ± 0.0020
ABTS [mM TE/g]	0.49	0.53 ± 0.0142	0.58	0.57 ± 0.0185

**Table 6:** Polyphenols profile of extracts obtained on the central point (Sample MAE-CP) and under the optimal conditions (Sample MAE-OPT).

Retention Time [min]	Compound	Sample MAE-CP	Sample MAE-OPT
		Measured Mass [m/z]/Error [mDa]	
12.67	Monogalloyl-glucose	331.07/0.48	331.07/0.58
14.14	Gallic acid	169.01/0.01	169.01/0.46
16.15	Vanillic acid	167.03/0.14	167.03/−0.04
19.28	Protocatechuic acid	153.02/0.12	153.02/0.32
37.61	3-*p*-Coumaroylquinic acid	337.09/0.92	337.09/0.92
37.61	4-*p*-Coumaroylquinic acid	337.09/0.92	337.09/0.92
39.33	(+)-Catechin	ND	289.07/0.69
39.33	(−)-Epicatechin	ND	289.07/0.69
39.53	Coumaric acid hexoside isomer-1	325.09/0.30	325.09/0.30
39.53	Coumaric acid hexoside isomer-2	325.09/0.30	325.09/0.30
39.53	Coumaric acid hexoside isomer-3	325.09/0.30	325.09/0.30
39.53	*p*-Coumaric acid	163.04/−0.19	163.04/−0.19
43.32	Dihydroxycoumarin	177.02/0.36	177.02/−0.07
44.46	Caffeic acid	179.03/0.13	179.03/0.05
52.08	*cis*-Coutaric acid	295.05/0.17	ND
52.08	*trans*-Coutaric acid	295.05/0.17	ND
66.28	Quercetin hexoside isomer-1	463.09/−0.14	463.09/0.98
66.28	Quercetin hexoside isomer-2	463.09/−0.14	463.09/0.98
66.28	Quercetin-3-*O*-galactoside	463.09/−0.14	463.09/0.98
66.28	Quercetin-3-*O*-glucoside	463.09/−0.14	463.09/0.98
66.61	Kaempferol-3-rutinoside	593.15/−0.11	593.15/−0.22
72.90	Naringenin-7-*O*-glucoside	433.11/−0.01	433.11/0.34
76.06	Quercetin-3-*O*-rutinoside	609.15/−0.09	609.15/0.01
76.37	Quercetin glucuronide	477.07/1.37	477.07/1.50
76.45	Kaempferol-3-galactoside	447.09/−0.59	447.09/0.03
76.45	Kaempferol-3-glucoside	447.09/−0.59	447.09/0.03
76.52	Isorhamnetin-3-*O*-galactoside	477.10/−0.64	477.10/−0.13
76.83	Eriodictyol	287.06/0.57	287.06/0.68
77.27	Naringenin	ND	271.06/1.42
78.10	Kaempferol	285.04/0.24	285.04/0.45
78.10	Luteolin	285.04/0.24	285.04/0.45
78.74	Quercetin pentoside isomer-1	433.08/0.30	ND
78.74	Quercetin pentoside isomer-2	433.08/0.30	ND
78.84	Quercetin	301.03/−1.45	ND
78.90	Ellagic acid	301.00/−0.80	301.00/−0.70

ND = not detected.

## Data Availability

Data are contained within the article or Supplementary Materials.

## Supplementary Materials

The following supporting information can be downloaded at: https://www.mdpi.com/article/10.3390/foods11091184/s1, Figure S1: Influence of (a) ethanol concentration, (b) extraction time, (c) liquid–solid ratio and (d) irradiation power on Y; Figure S2: Influence of (a) ethanol concentration, (b) extraction time, (c) liquid–solid ratio and (d) irradiation power on TP; Figure S3: Influence of (a) ethanol concentration, (b) extraction time, (c) liquid–solid ratio and (d) irradiation power on DPPH; Figure S4: Influence of (a) ethanol concentration, (b) extraction time, (c) liquid–solid ratio and (d) irradiation power on FRAP; Figure S5: Influence of (a) ethanol concentration, (b) extraction time, (c) liquid–solid ratio and (d) irradiation power on ABTS; Table S1: Summary of active networks and their training and test performances title.
