# Supplementary material for: Polyphenols Recovery from Thymus serpyllum Industrial Waste Using Microwave-Assisted Extraction–Comparative RSM and ANN Approach for Process Optimization
Source: Foods. 2022 Apr 19;11(9):1184. doi: 10.3390/foods11091184 (PMC9101683; doi:10.3390/foods11091184)
Supplement: Supplementary file 1 [file foods-11-01184-s001.zip › foods-1674586-supplementary.pdf]

Supplementary Materials

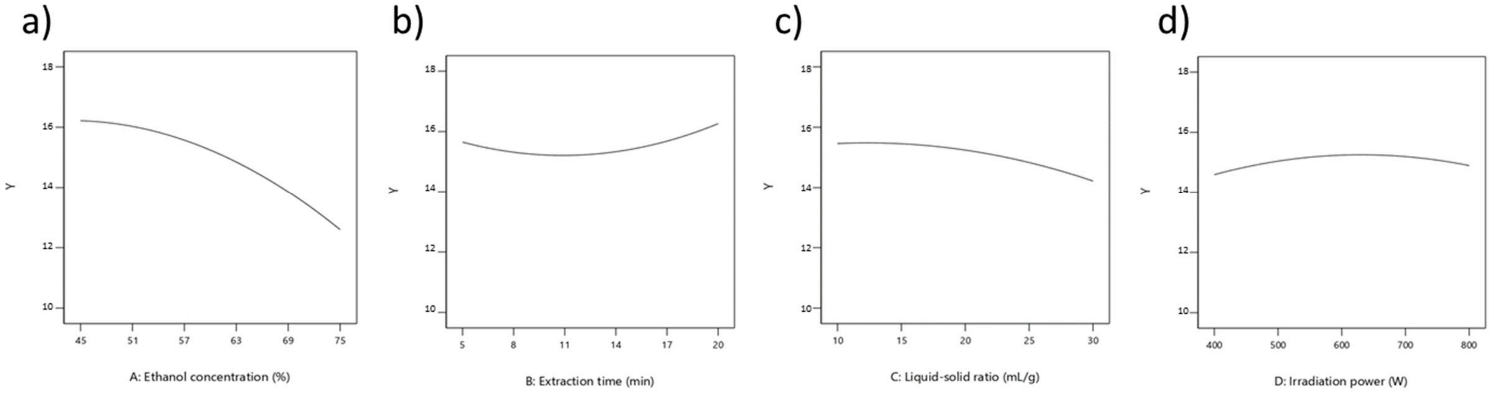

Figure S1. Influence of a) ethanol concentration, b) extraction time, c) liquid-solid ratio and d) irradiation power on Y

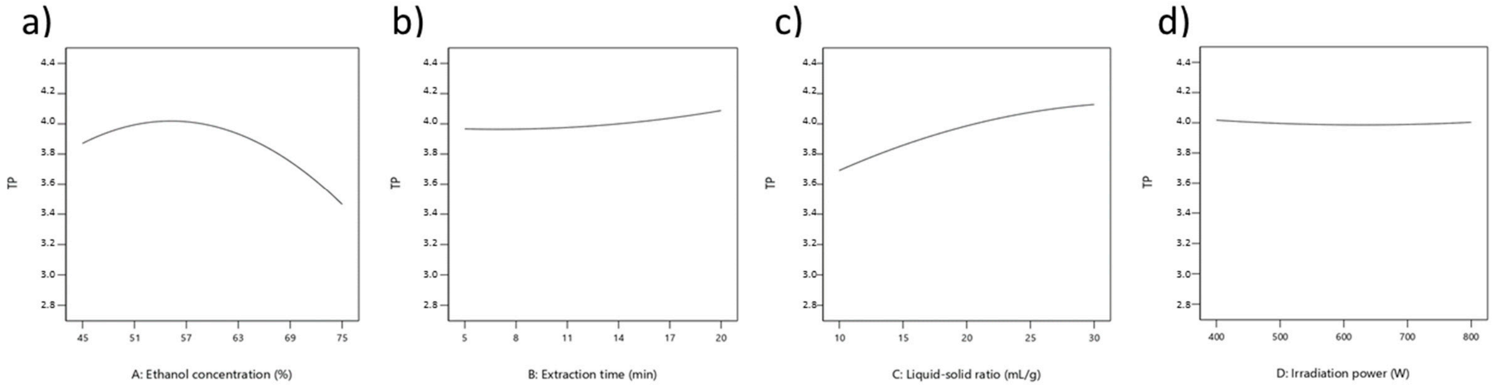

Figure S2. Influence of a) ethanol concentration, b) extraction time, c) liquid-solid ratio and d) irradiation power on TP

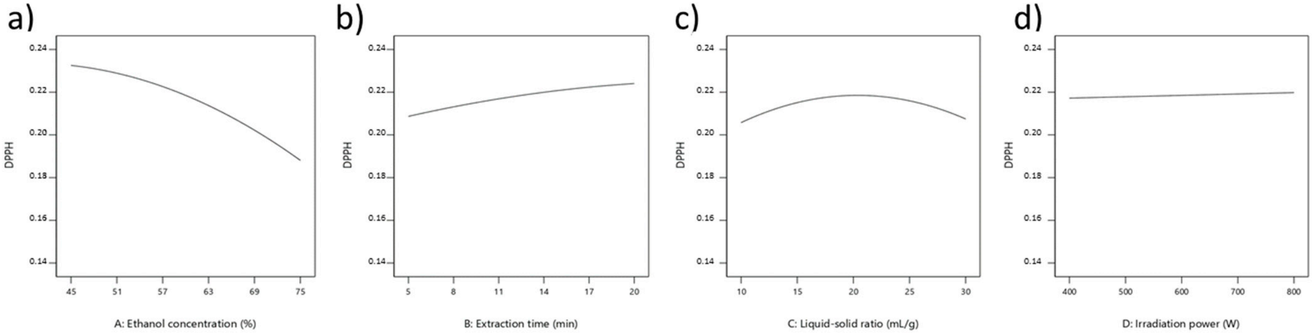

Figure S3. Influence of a) ethanol concentration, b) extraction time, c) liquid-solid ratio and d) irradiation power on DPPH

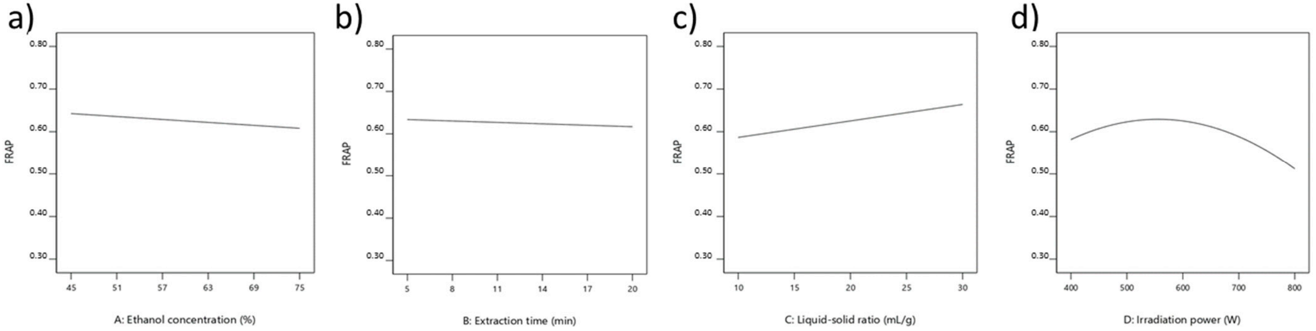

Figure S4. Influence of a) ethanol concentration, b) extraction time, c) liquid-solid ratio and d) irradiation power on FRAP

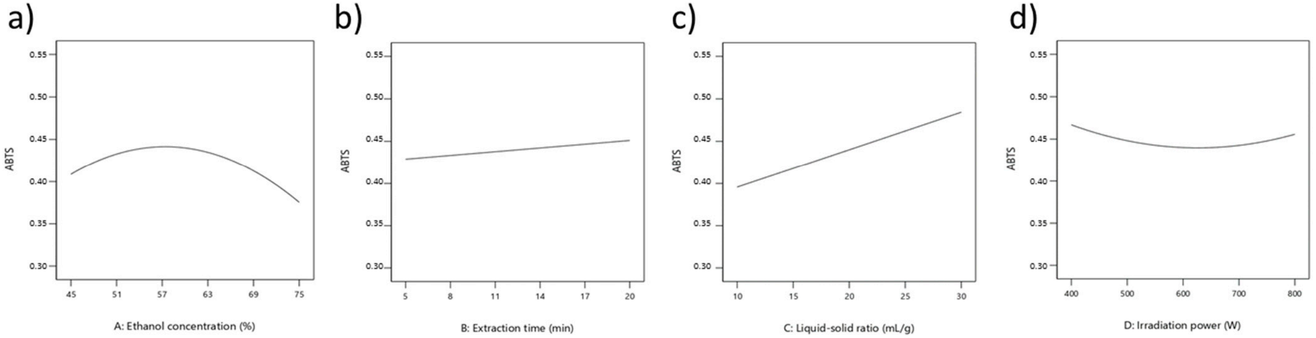

Figure S5. Influence of a) ethanol concentration, b) extraction time, c) liquid-solid ratio and d) irradiation power on ABTS

Table S1. Summary of active networks and their training and test performances

N. N. – number of neurons in the hidden layer

Response	N. N.	Training perf.	Test perf.	Validation perf.	Training error	Test error	Validation error	Training algorithm	Error function	Hidden activation	Output activation
Y	5	0.9510	0.9839	0.9917	0.0031	0.0036	0.0012	BFGS 19	SOS	Tanh	Logistic
TP	6	0.9610	0.9249	0.6409	0.0316	0.0421	0.1683	BFGS 23	SOS	Tanh	Tanh
DPPH	9	0.9660	0.9901	0.7766	0.0027	0.0034	0.0075	BFGS 50	SOS	Exponential	Logistic
ABTS	10	0.9600	0.9651	0.4795	0.0000	0.0001	0.0001	BFGS 25	SOS	Exponential	Logistic
Total	8	0.8968	0.7491	0.6432	0.0312	0.0432	0.1382	BFGS 15	SOS	Tanh	Exponential
